# Interventions to prevent obesity in Latinx children globally: protocol for a systematic review and meta-analysis

**DOI:** 10.1186/s13643-021-01674-w

**Published:** 2021-04-22

**Authors:** Rachel Bleiweiss-Sande, Arturo Jiménez-Cruz, Montserrat Bacardí-Gascón, Kara Skelton, Sara E. Benjamin-Neelon

**Affiliations:** 1 Department of Health, Behavior and Society, Johns Hopkins Bloomberg School of Public Health, 624 N Broadway, HH904, Baltimore, MD 21205 USA; 2 Department of Medicine and Psychology, Universidad Autónoma de Baja California, Universidad 14418, UABC, Parque Internacional Industrial Tijuana, 22390 Tijuana, B.C. Mexico; 3 Department of Health, Behavior and Society, Johns Hopkins Bloomberg School of Public Health, 615 North Wolfe Street, Suite W3041, Baltimore, MD 21205 USA

**Keywords:** Adiposity, Adolescent, Body mass, Children, Hispanic, Intervention, Latina, Latino, Overweight

## Abstract

**Background:**

Rates of childhood obesity have accelerated rapidly over the past decade in low- and middle-income countries and in Latin America in particular. At the same time, Latinx children in high-income countries have been disproportionately affected by obesity. Public health and medical experts have called for greater focus on multi-sector approaches to obesity prevention, including policy, systems, and environmental strategies, but current evidence for effective intervention strategies among Latinx children is lacking. Several systematic reviews have investigated obesity prevention interventions in Latinx children in the United States and in Latin America, including our own review, but these are now a decade old. Thus, an updated review of existing interventions is needed. To address this gap, we will conduct a systematic review and summary of interventions for obesity prevention among Latinx children published over the past 10 years. The objective of this paper is to outline the protocol for conducting the systematic review and possible meta-analysis.

**Methods:**

We will conduct a literature search using PubMed, ERIC, PsycINFO, Scopus, Scientific Electronic Library Online, and Google Scholar databases for studies of interventions to prevent obesity in Latinx children ages birth to 18 years of age. To meet our definition of an intervention, we will include study designs that evaluate the either the efficacy or effectiveness of obesity prevention interventions, including randomized controlled trials, quasi-experimental studies, and non-randomized interventions with a control or comparison group. We will exclude interventions that aimed to treat rather than prevent overweight or obesity. Interventions may take place in any country or setting. The primary outcome of interest will be child overweight or obesity, measured as adiposity, body mass, or similar anthropometric measures. We will assess risk of bias of included studies using the Cochrane risk of bias tool for randomized and non-randomized studies, as appropriate. We may conduct meta-analyses if studies with comparable exposure and outcome variables are available.

**Discussion:**

This protocol paper establishes a methodology for a future systemic review of obesity prevention interventions in Latinx children. A systematic review of this topic will provide an important update to the literature regarding interventions to prevent obesity in Latinx child populations globally over the past decade. Review results will be relevant to stakeholders across multiple sectors engaged in childhood obesity prevention among Latinx children.

**Systematic review registration:**

PROSPERO CRD42020161339

**Supplementary Information:**

The online version contains supplementary material available at 10.1186/s13643-021-01674-w.

## Background

Rates of childhood obesity have accelerated rapidly over the past decade in low- and middle-income countries and in Latin America in particular [1, 2]. Approximately 42.5 million children (about 20%) in Latin America had overweight or obesity in 2008–2013, and current rates may be higher [3]. Even more concerning, children in Latin America have experienced one of the fastest increases in age-standardized body mass index (BMI) globally [2]. Children in Latin America and the Caribbean now rank among the highest globally in terms of mean BMI, on par with those of high-income countries [2]. Given the precipitous rise in Latinx children with obesity, prevention in this population remains a critical public health priority.

In addition to the rising trends in children with obesity in Latin America and the Caribbean, Latinx children are disproportionately affected by obesity in high-income countries such as the United States (US). According to data from 2011 to 2012, nearly 40% of Latinx children in the US had overweight or obesity, representing the highest rates across all ethnic and racial groups [4]. Latinxs are one of the fastest growing populations in the US, reaching nearly 60 million in 2018 [5]. Although studies have demonstrated that recent Latinx immigrants to the US experienced lower rates of chronic disease compared to their non-Latinx white peers, more time spent in the US has been associated with having obesity [6].

The major drivers of obesity among Latinx children are diverse. Researchers have documented a shift in dietary intake and energy expenditure, referred to as the global nutrition transition, that has resulted in rapid increases in non-communicable diseases in less developed regions such as Latin America [7]. The consequences of this transition include rapid dietary changes, particularly increases in sugar-sweetened beverages [8, 9], animal product and processed food consumption [9, 10], and a concomitant decrease in vegetable and legume intake [8–10]. The nutrition transition has also resulted in changes in the local food environment, such as greater proximity to supermarkets and fast food outlets [11], and increased exposure to food advertising [12, 13], which play an important role in increasing rates of obesity among Latinx children. In addition, Latinx children have decreased physical activity and increased sedentary behaviors due to factors such as changes in transportation networks [14].

The local context within individual countries is diverse and complex, resulting in variable environmental and demographic influences on obesity outcomes among Latinx children [15]. Overweight and obesity varies by ethnicity (e.g., indigenous versus non-indigenous) [15], geographic location (e.g., urban versus rural residence) [16], socioeconomic status [17], and maternal education [15]. For example, there is evidence that Honduras and Peru experienced a higher prevalence of overweight and obesity in urban compared to rural areas, while the opposite was true in Bolivia and Nicaragua [15]. Despite these differences, the shared economic ties, common health threats, and increasing immigration between regions such as Latin and North America have prompted researchers to call for global responses to help prevent obesity among Latinx populations [18]. In particular, public health officials have endorsed policy, systems, and environmental strategies for obesity prevention to influence individual behaviors within broader social and environmental contexts [19, 20].

To date, two systematic reviews have summarized interventions to prevent obesity among Latinx children in the US [21, 22], and only one systematic review has summarized interventions in the US and Latin America through 2010 [23]. Importantly, these reviews identified few studies that targeted policy, systems, and environmental intervention strategies for obesity prevention [23]. In recent years, several obesity prevention interventions aimed at Latinx children have been published, with mixed results [24–30]. Given the continuing rise in the number of children with obesity, there is a need to update the literature to identify and highlight successful interventions. To our knowledge, no study has systematically investigated interventions to prevent obesity among Latinx child populations globally over the past decade. As such, we will conduct a systematic review and possible meta-analysis of interventions for obesity prevention among Latinx children. The objectives of this systematic review are to answer the following research questions:

1. How many obesity prevention interventions in Latinx child populations have been conducted and in what countries and settings?
2. How many obesity prevention interventions in Latinx child populations have been effective and to what degree?
3. Of effective interventions, what strategies (e.g., policy, systems, environmental) were used?
4. Does the effectiveness of obesity prevention interventions vary by sociodemographic factors of children or families (e.g., geographic location, gender, family income, parental education)?
5. What are the major knowledge gaps, if any, that exist regarding obesity prevention interventions among Latinx children?

In this paper, we aim to outline the protocol for a future systematic review of obesity prevention interventions in Latinx children.

## Methods

### Registration

This protocol is registered with the PROSPERO database for systematic reviews (#CRD42020161339) and reported according to the Preferred Reporting Items for Systematic Review and Meta-Analysis Protocols (PRISMA-P) [31] guidelines (Additional file 1).

### Eligibility criteria

We will conduct a systematic review of interventions aimed at preventing obesity in Latinx children. We will use the population, intervention, comparison, outcome, and study design (PICOS) framework to guide our study selection as outlined below [32].

#### Population

We will select studies that include child populations (age 0–18) with a minimum of 50% of participants identified as Latinx. The term Latinx is used to describe the ethnicity of Mexican, Central American, South American, and Caribbean origin individuals and those of Latin-American descent living in the United States (US) and in other countries [33]. For interventions taking place in Latin-American countries and Spanish-speaking countries in the Caribbean, we will assume that the majority of participants are Latinx, unless stated otherwise. If a potentially eligible study does not describe the ethnic background of participants, we will contact the study authors to request complete demographic information. Interventions aimed at mothers during pregnancy will be excluded.

#### Intervention

This review will include primary research evaluating interventions that aim to prevent childhood obesity. These may include interventions that target obesogenic behaviors or risk factors for obesity, including diet, physical activity, sedentary behavior, screen time exposure, stress, and sleep — or any combination of these behaviors. We will exclude studies evaluating the effectiveness of interventions to treat childhood obesity, including pharmacological or surgical studies.

#### Comparison

We will only include studies with a control or comparison group, such as a no-intervention control or comparison group, alternative-intervention control or comparison group, or delayed-intervention control or comparison group. We will also include studies with multiple intervention groups, such as a diet-only group, a diet plus physical activity group, and a comparison group. We will exclude studies without a control or comparison condition.

#### Outcomes

The primary outcome of interest is change in obesity, measured by adiposity or body mass, body mass index, age- and sex-standardized body mass index, fat mass, skinfold thickness, waist or hip circumference, or other anthropometric measures. We will include only those studies using direct measurement by researchers, study staff, or parents, and will exclude studies with only self-reported measures of obesity.

#### Study design

We will include study designs that involve evaluation of policy, systems, and environmental change strategies to prevent childhood obesity, such as natural experiments. Specifically, we will include study designs that evaluate the efficacy or effectiveness of obesity prevention interventions, including randomized controlled trials, quasi-experimental studies, and non-randomized interventions.

### Search methods

We will conduct a literature search using PubMed, ERIC, PsycINFO, Scopus, Scientific Electronic Library Online (SciELO), and Google Scholar databases. We developed initial search terms using a combination of Medical Subject Headings (MeSH) and keyword terms for obesity prevention interventions among Latinx children and informed by search strategies used in related systematic reviews [21–23]. We have provided an example search strategy developed for PubMed (Additional file 2). To account for differences in controlled vocabulary and syntax rules, we will revise the search strategy for each database. We will perform forward and backward citation searches of included studies to look for additional publications. We may repeat database searches prior to final analyses to capture any recently published intervention studies in our review.

This review will build on the findings of previous systematic reviews of obesity prevention interventions in Latinx children in the US [21, 22] and Latin America and the US [23]. These reviews were restricted to studies published before 2010 [21, 23] and 2012 [22]. Therefore, we will include studies published from January 2010 to July 2020 to provide a comprehensive review of the most recent interventions conducted over the past decade in Latinx children globally. We will not impose language restrictions on studies. For non-English language studies, we will obtain translations of key sections or published papers in their entirety via members of the research team for Spanish language studies, or using the Cochrane Task Exchange [34] for other language studies, as needed.

### Study records

#### Data management

We will use Covidence Software (Covidence Systematic Review Software, Veritas Health Innovation, Melbourne, Australia), an online tool for systematic review management, to manage the review process. Covidence manages study screening, study selection, and data abstraction. Covidence also automatically de-duplicates citation records based on title, date, and author matches.

#### Selection process

We will assess studies for inclusion using a two-step review process. First, two authors will independently screen titles and abstracts for eligibility against the following criteria:

1. Age of children at baseline: are participants > 0 years but < 18 years?
2. Population race or ethnicity: is at least 50% of the study population Latinx?
3. Study design: is the study design a randomized or non-randomized trial, quasi-experimental trial, or non-randomized intervention?
4. Comparator group: is there a control or comparison group?
5. Exposure: was an intervention to prevent childhood obesity investigated as the main exposure, not including a pharmacological or surgical intervention?
6. Outcomes: was a measure of obesity, such as adiposity or body mass index, investigated as an outcome?
7. Reporting method: was the obesity outcome obtained and reported through direct measurement by researchers, study staff, or parents?
8. Publication date: was the study published on or after January 1, 2010?

We will retrieve full-text manuscripts of studies marked as “yes” or “not clear” to all eligibility questions by either study author. If we are unable to obtain a full text for screening after exhausting all possible efforts (e.g., inter-library loan, contacting authors), we will exclude the article.

For the second phase of the screening process, two authors will carry out full-text screening using the eligibility criteria outlined above. We will contact study authors to clarify eligibility questions and request missing information. We will include studies that meet all inclusion criteria and do not meet any exclusion criteria. We will resolve eligibility disagreements first through discussion among two reviewers and with a third reviewer if necessary. Both reviewers will document reasons for exclusion at the full-text review stage within Covidence, which will be used to generate a PRISMA flow diagram.

#### Data extraction

We will extract the following information for quality assessment and evidence synthesis using a pre-piloted form within Covidence: publication details (study ID, PubMed ID, journal, title, authors, language), country, study design (e.g., randomized control trial, quasi-experimental trial), intervention details (setting, content, format, delivery, control or comparison group), baseline participant demographics and characteristics (e.g., geographic location, gender, family income, parental education), recruitment and intervention completement rates, weight-related outcomes, statistical methods (covariates used, amount of missing data), results, and limitations.

#### Quality assessment

To assess bias, we will use the Cochrane Collaboration’s risk of bias tool (ROB) for randomized [35] or non-randomized studies (ROBINS-I) [36], as appropriate. The Cochrane ROB tool is used to rate studies according to their level of bias (high, low, or unclear) across seven domains: (1) random sequence generation, (2) treatment allocation concealment, (3) blinding of participants and personnel, (4) blinding of outcome assessment, (5) completeness of outcome data, (6) selective outcome reporting, and (7) other sources of bias [35]. The ROBINS-I tool also covers seven distinct domains through which bias may be introduced, including (1) confounding, (2) selection of participants into the study, (3) classification of interventions, (4) deviations from intended interventions, (5) missing data, (6) measurement of outcomes, and (7) selection of the reported result [36]. Using the ROBINS-I, we will rate studies as having low, moderate, serious, or critical risk of bias, or not enough information [36]. Two reviewers will independently assess risk of bias for included studies. Any disagreements will be discussed and resolved by a third reviewer.

We will summarize the quality of included studies using the Grading Quality of Evidence and Strength of Recommendations (GRADE) approach [37]. GRADE rates the quality of studies as high, moderate, low, or very low across four areas, including methodological flaws, consistency of results across studies, generalizability to the target population, and effect size [38]. Two reviewers will independently assess study quality, with disagreements resolved by a third reviewer.

### Evidence synthesis

We will create a table to synthesize study characteristics and obesity outcomes. We will present results by country, age group (e.g., 0–5 years, 6–12 years, 13–18 years), and setting (e.g., early care and education, community, primary school, primary care) as appropriate. We will provide a narrative synthesis of the review results, organized by intervention design; measure of obesity used; country (low-income, middle-income, high-income), and sociodemographic characteristics (e.g., household income and urban versus rural residence). We will highlight any gaps in available evidence for obesity prevention interventions among Latinx children, if present, according to the characteristics outlined above.

If studies with comparable exposure and outcome variables are available, we may perform separate meta-analyses for each group of studies. Assuming that studies are comparable and that we have access to the relevant data, we will use fixed-effect and random-effect models, as appropriate, to calculate pooled effects sizes among comparable studies. Intervention strategies aimed at obesity prevention are likely to differ substantially depending on the age group targeted due to the developmental changes that occur throughout childhood. Therefore, we may perform sub-analyses by age group or setting, as warranted by the available studies. We may also explore the homogeneity of effects among studies using forest plots, Cochrane’s *Q* test (chi-squared), and Higgins *I*^2^ statistics [39]. For studies with *I*^2^ values greater or equal to 50% (indicative of average or above average heterogeneity) [39], we may perform additional sub-group analyses according to the exposure variables outlined above. To test the presence or absence of publication bias, we will use funnel plots to visually inspect for asymmetry, and Egger’s regression test [40] for quantitative evidence of bias if appropriate. We will report findings of homogeneity and publication bias in the results and elaborate on the implication of these findings in the discussion.

### Amendments to protocol

We will register any amendments to this protocol with PROSPERO and document all changes in the final publication.

## Discussion

In this paper, we outline a protocol for conducting a systematic review and possible meta-analysis of obesity prevention interventions among Latinx children, including the rationale for selection and use of appropriate tools and strategies to manage and conduct the review. This systematic review and potential meta-analysis will be the first to examine the efficacy and effectiveness of obesity prevention interventions among Latinx children globally over the past decade. We may conduct secondary analyses to examine differences in obesity outcomes according to study and participant characteristics, such as geographic location, child age, child gender, study setting, and quality of studies. By evaluating the impact of interventions on obesity-related outcomes, we hope to identify the most effective interventions and their policy, systems, and environmental strategies for obesity prevention in Latinx children. We will also identify potential gaps in the literature to inform future intervention efforts.

This review will build on the findings of previous systematic reviews and also provide an important update and global perspective on interventions for obesity prevention in Latinx children. Of the previously published systematic reviews on this topic, two focused on interventions conducted in the US [21, 22], and one investigated interventions in the US and Latin America [23]. These reviews identified several effective school-based obesity prevention interventions in the US and Latin America, and demonstrated the effectiveness of including parent components in school-based interventions [21–23]. Several successful primary care-based interventions were highlighted in Latin America; however, effect sizes were small [23]. Importantly, these reviews examined studies published prior to 2010 [21, 23] and 2012 [22], and included only those published in English. In addition, no systematic review has included interventions conducted in Latinx populations in regions outside of the US and Latin America, such as the Caribbean, where childhood obesity rates are similar to that of Latin-American countries [41]. This systematic review will address these gaps by providing an update of the literature published since 2010. We will include non-English language studies if available, as well as interventions conducted in Latinx child populations in any country or setting. These steps will allow us to provide a more complete synthesis of current evidence on this topic.

This review will include interventions that use policy, systems, and environmental strategies for obesity prevention. These approaches have emerged as effective strategies to address complex public health issues, such as obesity prevention in children [19, 42]. Rather than focus on individual determinants of obesity, policy, systems, and environmental strategies attempt to influence the broader social and environmental context to support diet and physical activity changes [19]. These approaches are particularly important for low-income and racial and ethnic minority populations that are at greater risk for obesity, such as Latinx children, since they address underlying determinants of health and social inequity [20, 43]. The World Health Organization has endorsed integrated and multi-sector interventions to prevent obesity in children, particularly in low- and middle-income countries, that recognize related issues such as food security and undernutrition [44]. Policy, systems, and environmental strategies may be implemented in a variety of sectors and settings. For example, dietary behaviors may be influenced through nutrition education in early care and education and school settings, food distribution in community environments, or taxation at the national level. Understanding the nature and efficacy of strategies that have been implemented for obesity prevention among Latinx children is critical to advancing successful approaches on a broader scale [19, 44].

We acknowledge there may be several limitations to this review. First, by including only studies with a measure of obesity as an outcome, we may be overlooking promising intervention strategies that measure secondary outcomes, such as changes in diet, physical activity, or sleep — behaviors related to obesity. However, interventions that address specific behaviors, such as sugar-sweetened beverage intake [45] and physical activity [46, 47], have been reviewed and summarized elsewhere. Second, we anticipate that the intervention strategies, settings, and outcome measures used may vary across studies. To address this, we will attempt to group studies according to relevant characteristics, such as intervention strategy, setting, or child age. We may then provide a narrative review of the included studies by relevant characteristics. Third, it is possible that in conducting meta-analyses, we may introduce meta-bias into our review [48]. We will take steps to address this issue, including examination of funnel plots, and completing a quality of evidence synthesis using the GRADE approach. Finally, our review may be restricted by the number of interventions for obesity prevention with comparable exposure and outcomes variables, thus limiting our ability to conduct meta-analyses. Regardless, we will present a tabular synthesis of included studies and their outcomes.

This protocol paper details the methodology for conducting a future review of obesity prevention interventions in Latinx children. The protocol described will also serve as guidance for researchers that aim to implement future systematic reviews on similar topics. Ultimately, the systematic review resulting from this protocol will provide an important update to the literature regarding interventions to address obesity in Latinx child populations globally over the past decade. Given that rates of obesity are increasing rapidly among this population of children in both high-income and developing countries, preventing obesity among Latinx children is an issue of critical global public health importance. Results from this review will help to highlight effective obesity prevention strategies in a vulnerable population of children. This review may also shed light on gaps in the literature to inform directions for future intervention research. Results will be relevant to stakeholders across multiple sectors engaged in childhood obesity prevention in Latinx children.

## Supplementary Information

**Additional file 1.** PRISMA-P 2015 Checklist

**Additional file 2.** Sample search strategy for PubMed

## Data Availability

Not applicable.

## Abbreviations

GRADE: Grading Quality of Evidence and Strength of Recommendations
PRISMA: Preferred Reporting Items for Systematic Review and Meta-Analysis Protocols
ROB: Risk of bias
ROBINS-I: Cochrane Collaboration’s risk of bias tool for non-randomized studies
US: United States
