# Supplementary material for: Interventions to prevent obesity in Latinx children globally: protocol for a systematic review and meta-analysis
Source: Syst Rev. 2021 Apr 22;10:123. doi: 10.1186/s13643-021-01674-w (PMC8063476; doi:10.1186/s13643-021-01674-w)
Supplement: Supplementary file 2 — Additional file 2. Sample search strategy for PubMed [file 13643_2021_1674_MOESM2_ESM.docx]

**Additional File 2**

**Sample search strategy for PubMed**

| #1 | intervention[Title/Abstract] OR approach*[Title/Abstract] OR strateg*[Title/Abstract] OR program*[Title/Abstract] OR trial[Title/Abstract] OR "program evaluation"[Title/Abstract] OR "prevention and control"[Subheading] OR "early intervention, educational"[MeSH] OR "randomized controlled trials as topic"[MeSH] OR "non randomized controlled trials as topic"[MeSH] OR "Controlled Clinical Trial"[Publication Type] OR "Evaluation Study"[Publication Type] OR "Randomized Controlled Trial"[Publication Type] |
| --- | --- |
| #2 | "behavior change"[Title/Abstract] OR “behavior modification”[Title/Abstract] OR behavior*[Title/Abstract] OR prevent*[Title/Abstract] OR "healthy eating"[Title/Abstract] OR "physical activity"[Title/Abstract] OR exercise[Title/Abstract] OR fitness[Title/Abstract] OR “health education"[MeSH] OR "Feeding Behavior"[MeSH] OR "Family Health"[MeSH] OR "Health Promotion"[MeSH] OR "Diet, Healthy"[MeSH] OR "Recreation"[MeSH] OR "Motor Activity"[MeSH] OR "Motor Skills"[MeSH] OR "Exercise"[MeSH] OR "screen time"[MeSH] OR "sleep"[MeSH] OR "Parents/education"[MeSH] |
| #3 | infan*[Title/Abstract] OR newborn*[Title/Abstract] OR baby[Title/Abstract] OR babies[Title/Abstract] OR child*[Title/Abstract] OR teen*[Title/Abstract] or adolescen*[Title/Abstract] OR youth[Title/Abstract] OR pre-school*[Title/Abstract] OR preschool*[Title/Abstract] OR preschool-age*[Title/Abstract] OR "pre-school age*"[Title/Abstract] OR toddler*[Title/Abstract] OR "nursery school*"[Title/Abstract] OR kinder*[Title/Abstract] OR pediatric[Title/Abstract] OR paediatric[Title/Abstract] OR “middle school*”[Title/Abstract] OR "high school*"[Title/Abstract] OR “secondary school” [Title/Abstract] OR “primary school*”[Title/Abstract] OR “lower school”[Title/Abstract] OR “elementary school”[Title/Abstract] OR afterschool[Title/Abstract] OR “out-of-school” [Title/Abstract] OR “summer school” [Title/Abstract] OR "Adolescent"[MeSH] OR "child"[MeSH] OR “infant"[MeSH] OR "Child, Preschool"[Mesh] OR "Child Day Care Centers"[Mesh] OR "Schools, Nursery"[MeSH] |
| #4 | obes*[Title/Abstract] OR overweight[Title/Abstract] OR "child* obesity"[Title/Abstract] OR BMI[Title/Abstract] OR "BMI z*"[Title/Abstract] OR "BMI-z*"[Title/Abstract] OR "body mass*"[Title/Abstract] OR "healthy weight"[Title/Abstract] OR "obesity prevention"[Title/Abstract] OR "weight status"[Title/Abstract] OR "weight-for-length"[Title/Abstract] OR "weight for length"[Title/Abstract] OR metaboli* [Title/Abstract] OR "Pediatric Obesity/prevention and control"[Mesh] OR "Pediatric Obesity/ethnology"[Mesh] OR "body weights and measures"[MeSH] OR "adiposity"[MeSH] OR "Body Mass Index"[MeSH] OR “Pediatric Obesity”[MeSH] OR "Overweight"[Mesh] OR "Overweight/ethnology"[Mesh] OR "Overweight/therapy"[Mesh] OR "Overweight/diagnosis"[Mesh] |
| #5 | Latin*[Title/Abstract] OR Hispan*[Title/Abstract] OR chican*[Title/Abstract] OR mexic*[Title/Abstract] OR divers*[Title/Abstract] OR “low*-income” [Title/Abstract] OR “low* income”[Title/Abstract] or urban[Title/Abstract] OR Spanish*[Title/Abstract] OR Portuguese*[Title/Abstract] OR "head start"[Title/Abstract] OR "early head start"[Title/Abstract] OR "Hispanic Americans"[MeSH] OR "Americas"[MeSH] OR "Socioeconomic Factors"[MeSH] OR "Minority Groups"[MeSH] OR "Urban Population"[MeSH] OR "Rural Population"[MeSH] OR "Poverty/ethnology"[MeSH] OR "Emigrants and Immigrants"[Mesh] |
| #6 | "Humans"[Mesh] |
| #7 | "Review" [Publication Type] OR "Meta-Analysis" [Publication Type] OR "Observational Study" [Publication Type] OR "Specialties, Surgical"[Mesh] OR "Pharmacology"[Mesh] OR "Cross-Sectional Studies"[Mesh] |
| #8 | #1 AND #2 AND #3 AND #4 AND #5 AND #6 NOT #7 |
| Limits: | Published on or after January 1, 2010 |
